# Successful treatment of severe pneumonia, pyopneumothorax with severe acute respiratory distress syndrome, and septic shock: a case report

**DOI:** 10.1186/s40001-020-00459-6

**Published:** 2020-11-11

**Authors:** Xi Wang, Jian Lan, Ruijie Zhang, Xiaoqing Luo

**Affiliations:** The Second Affiliated Hospital of Chongqing Medical University, Chongqing, China

**Keywords:** Severe pneumonia, Acute respiratory distress syndrome (ARDS), Septic shock, Continuous renal replacement therapy (CRRT), Mechanical ventilation

## Abstract

**Background:**

This article reports a patient who survived severe pneumonia, pyopneumothorax with acute respiratory distress syndrome (ARDS), and septic shock, which is very difficult to treat.

**Case presentation:**

Antibiotics, continuous renal replacement therapy (CRRT), bronchial lavage and other treatments were used to treat a patient with pneumonia, pyopneumothorax, severe ARDS and septic shock. After comprehensive treatment, the patient was successfully treated and survived for a long time.

**Conclusions:**

There is a low successful clinical treatment rate for patients with pneumonia, pyopneumothorax with severe ARDS and septic shock. The successful treatment of this patient benefited from early and effective empirical therapy, targeted drug selection in the later stage, adequate closed thoracic drainage, repeated bronchial lavage, early CRRT, an appropriate respiratory support mode and parameter setting, immunotherapy and nutritional support therapy. This paper proposes a reference diagnosis and treatment solution for similar cases.

## Background

According to recommendations from the Infectious Diseases Society of America/American Thoracic Society (IDSA/ATS), the main diagnostic criterion for severe pneumonia should be the need for invasive mechanical ventilation or the presence of vasoconstrictor-requiring septic shock. Severe pneumonia can induce ARDS. Severe ARDS can be diagnosed when the oxygenation index (OI) is lower than 100 mmHg (positive end expiratory pressure (PEEP) ≥ 5 cmH_2_O), leading to 46.1% hospital mortality [1]. For ARDS treated with mechanical ventilation, the incidence of pneumothorax is 6.5%, and the mortality rate in that case is 51.4% [2]. Severe pneumonia may lead to sepsis and life-threatening organ dysfunction. ARDS associated with sepsis has a higher mortality rate than that with nonsepsis [3].

At present, there are no clinical guidelines for the treatment of severe pneumonia, pyopneumothorax with severe ARDS and septic shock. Studies have shown that early initiation of CRRT is associated with favorable clinical outcomes in ARDS patients [4]. We will share a successful case here. The present patient was critically ill, with a rapid progression of his condition. He was discharged from the hospital after receiving comprehensive treatment, including early CRRT, and has survived to this day.

## Case presentation

**Fig. 1:**
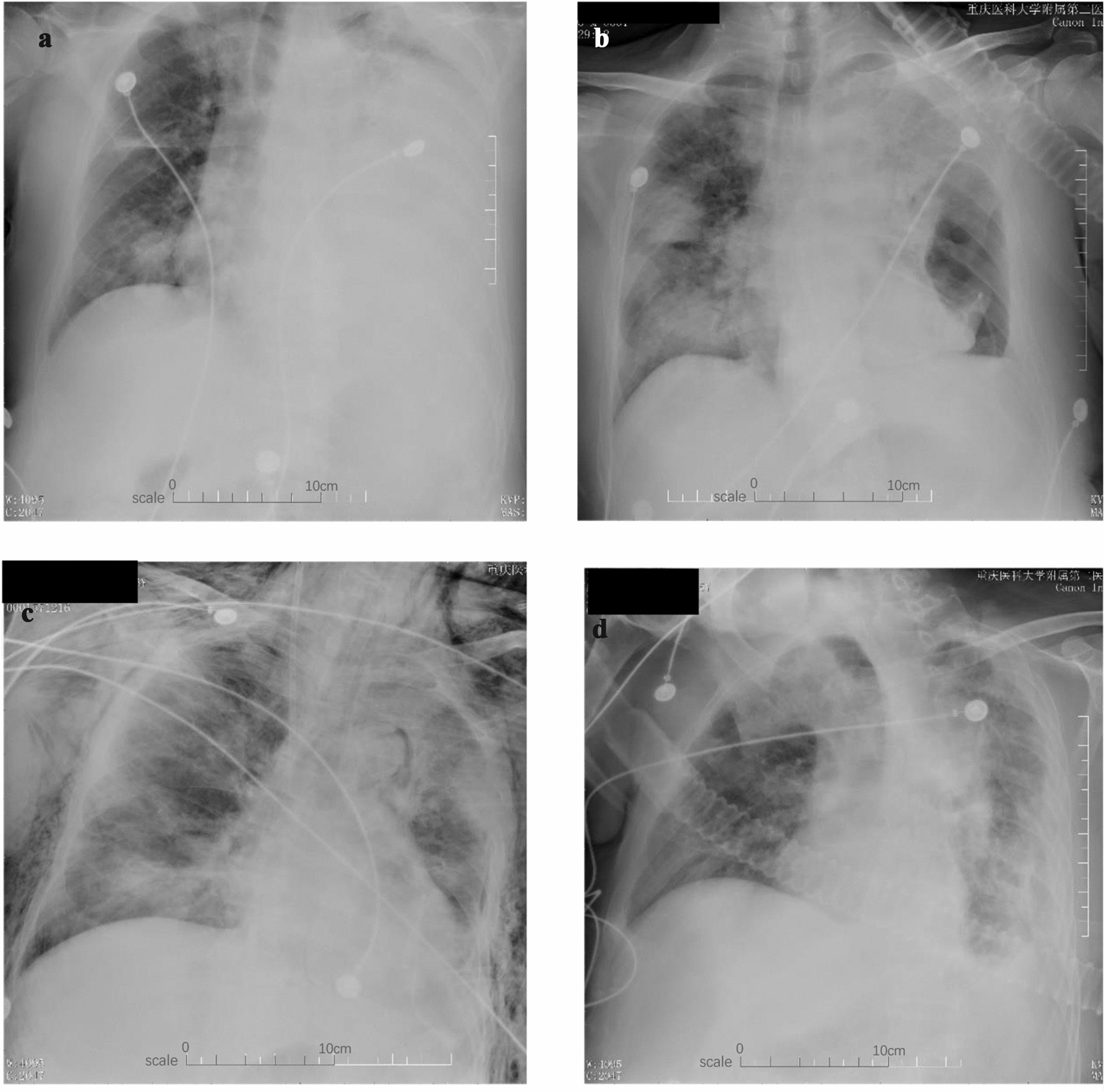
Series of bedside chest films: **a** A large quantity of left pleural effusions with inflammation of the right lung; **b** The left pleural effusion was partly absorbed while the encapsulated gas was added, and the shadow of the right lower lung mass became larger and thicker; **c** Pneumatosis occurred in the mediastinum, and an extensive accumulation of gas was found in both sides of the neck and under the skin of the chest wall; **d** The inflammation of both lungs was reduced, and the subcutaneous gas was basically absorbed

**Fig. 2:**
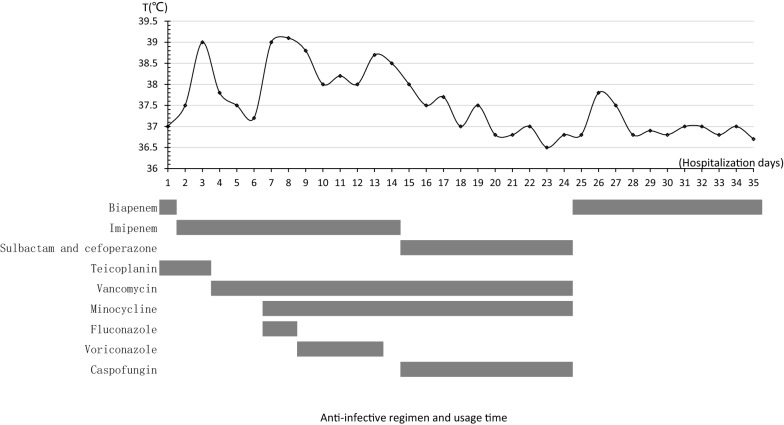
Changes in the body temperature and anti-infective regimen during hospitalization

A 65-year-old man was admitted to the respiratory intensive care unit (RICU) on December 15, 2018, after experiencing symptoms of fever, cough, and left chest pain for 10 days and dyspnea for 1 day. He had had hypertension, diabetes mellitus and diabetic nephropathy for many years and had controlled these conditions by taking medication. On admission, the patient showed high scores of 21, 4, and 6, respectively, on the Acute Physiology and Chronic Health Evaluation II (APACHE II), SOFA, and Geneva scales. His bedside chest film showed serious left pleural effusions with right lung inflammation (Fig. 1a), and rising inflammatory markers (white blood cells (WBC) 27.55*10^9^/L, procalcitonin (PCT) 2.8900 ng/ml and C-reactive protein (CRP) > 200 mg/L) with some other poor laboratory results (D dimer 0.5 mg/l, lactic acid 10.40 mol/L, albumin 23.4 g/L, creatinine 171.7 µmol/L, B-type natriuretic peptide precursor 1437.00 pg/mL). The patient was treated with carbapenems and glycopeptides (Fig. 2), the antibacterial spectrum of which covered Gram-positive bacteria, Gram-negative bacteria, *Pseudomonas* and anaerobes. At the same time, the patient was also treated with noninvasive ventilation (ST mode, FiO_2_ 90%, F 18 BPM, IPAP 13 cmH_2_O, EPAP 5 cmH_2_O), cardiotonic and sodium bicarbonate. A few hours later, the patient developed irritability, cool and moist limbs and shock. A 780 mL purulent liquid–gas mixture was extracted from his left chest cavity, and a thoracic drainage tube was placed into the left chest for continuous drainage. Examination of the pleural effusion showed that the total number of cells was 681,889*10^6^/L, and the number of nucleated cells was 245,589*10^6^/L. After these measures, the patient's dyspnea was slightly relieved.

This patient worsened from his second day of admission. He began to exhibit hypotension with respiratory distress. His OI dropped to 67 mmHg. The laboratory tests yielded worse indicators—WBC 38.63*10^9^/L, PCT 19.0700 ng/m, CRP > 200 mg/L, BNP 4711.00 pg/m and an estimated glomerular filtration rate (eGFR) decreased to 26 mL/min. The bedside chest film showed encapsulated pneumothorax on the left chest cavity (Fig. 1b). Endotracheal intubation and invasive mechanical ventilation were implemented (P-A/C mode, FiO2 100%, F 18/min, Pi 16 cmH_2_O, PEEP 4 cmH_2_O). At the same time, a sedative, an analgesic and a muscle relaxant were employed. Norepinephrine, low molecular weight heparin, enteral nutrition and parenteral nutrition support were also used. Bronchoalveolar lavage was performed to identify extensive erosion and edema of the bronchial mucosa and serious obstruction of the airway by copious purulent secretions. Daily bronchoalveolar lavage was carried out for the next 11 days to extubation.

**Fig. 3:**
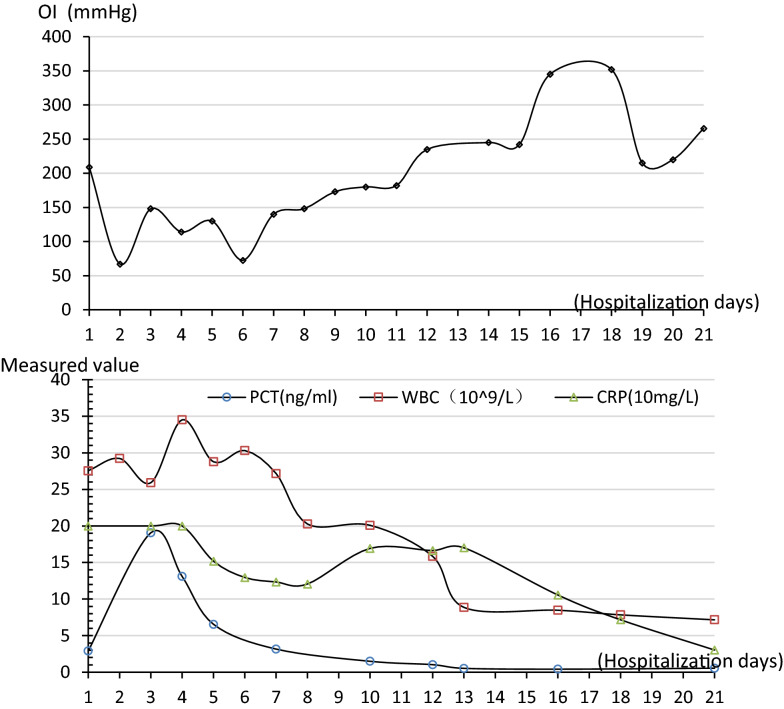
Changes in the oxygenation index and inflammatory factors

Bedside CRRT was performed for 8–12 h a day for 3 consecutive days from the third day of admission, and the ultrafiltration volume was 150 mL, 100 mL and 1116 mL, respectively. After CRRT, the patient maintained stable vital signs without using norepinephrine and muscle relaxants. From the 5th day of admission, gamma globulin was given at 20 g a day for 3 consecutive days, after which the OI of the patient increased gradually (Fig. 3). During the treatment, the sputum culture suggested yeast-like fungal growth, while multiple blood cultures did not indicate any positive results. *Stenotrophomonas maltophilia*, whose sensitivity test suggested that it was sensitive to minocycline, was found in the left pleural pus. *Aspergillus* was observed in the sputum of the endotracheal tube. *Aspergillus fumigatus* was present in the bronchoalveolar lavage fluid. Therefore, the treatment was supplemented with antifungal agents and minocycline.

On the 10th day, this patient developed subcutaneous emphysema with dyspnea (Fig. 1c). Considering that compression and obstruction of the drainage tube could inhibit improvement of this syndrome, the closed thoracic drainage tube was replaced. On the 12th day, the inflammatory markers continued to decrease, and the eGFR increased to 53.6 mL/min. Spontaneous breathing experiments (SBT) were performed for 2 h, and the rapid shallow breathing index (RSBI) was 80. Then, the endotracheal tube was removed, and the patient was switched to noninvasive mechanical ventilation. The thoracic drainage tube was removed on the 14th day, and the anti-infective regimens at that time were sulbactam, cefoperazone, vancomycin, minocycline and caspofungin. On the 17th day, the patient’s bedside chest film showed reduced inflammation and basically absorbed subcutaneous gas (Fig. 1d). Nasal catheters replaced noninvasive mechanical ventilation, and biapenem was used alone to resist infection on the 21st day. The patient discharged himself from the hospital after 35 days of admission and was satisfied with the treatment method and effect. He was followed-up at the outpatient clinic of the same hospital. He is presently leading a normal life, with his eGFR remaining at 60 mL/min (as of October 2020).

## Discussion and conclusions

The patient in the reported case had obvious respiratory distress and symptoms of infected, toxic blood. Imaging and pleural aspiration supported the diagnosis of severe pneumonia and pyopneumothorax. The patient reached the diagnostic criteria of severe ARDS and septic shock. Based on experience in severe pneumonia treatment, we have applied a wide range of anti-infection schemes and strengthened the antianaerobic therapy. In the follow-up treatment, we adjusted the antibiotics according to the positive bacteriological results and the drug sensitivity test. Effective antibacterial treatment was the key to improving the primary etiology and resolving the disease. The continuous drainage of pleural pus and repeated bronchoalveolar lavage also played an important role in the rapid and effective control of the infection. It has been reported that bronchoalveolar lavage is an effective treatment for mechanically ventilated patients with nosocomial pneumonia [5]. By observing multiple inflammatory indicators (Fig. 3) as monitored, the infection control measures were effective and appropriate.

Since the patient had diabetic nephropathy, we performed CRRT when his renal function began to decline, but creatinine levels did not significantly increase to protect his organs or improve the OI. It has been reported that during acute renal injury (AKI) in critically ill and elderly patients, early application of CRRT results in a better prognosis, and an eGFR lower than 30 mL/min/1.73 m^2^ is a strong risk factor for reducing long-term survival [6, 7]. This patient’s eGFR was reduced to 26 mL/min during treatment. He can survive for a long time, and his eGFR, which has been maintained at 60 mL/min during the follow-up after his discharge indicates the importance of early implementation of CRRT.

Pneumothorax is a serious complication of mechanical ventilation for patients with ARDS. The risk of pneumothorax developing into tension pneumothorax is high, and therefore, rapid drainage is needed [8]. When the patient in this case study was admitted, his chest film did not show hydropneumothorax; noninvasive ventilation was thought to have caused barotrauma, which was also one of the reasons for the patient’s rapidly deteriorating condition after admission. Timely and adequate closed thoracic drainage relieved the high intrathoracic pressure and created conditions for endotracheal intubation and invasive mechanical ventilation. During the treatment, a large amount of subcutaneous and mediastinal gas accumulation was aspirated from the blocked drainage tube. This also shows the importance of adequate thoracic drainage for patients with pneumothorax during mechanical ventilation. The choice of the mechanical ventilation mode and parameters is an important factor affecting the incidence of barotrauma. There is a particularly high risk when the end-inspiratory platform pressure is above 35 cmH_2_O [9]. Since mechanical ventilation is not conducive to healing pleural lacerations, the ventilator parameters should be set to a principle of a low PEEP and a low tidal volume with the prerequisite of an ensured OI.

Sepsis can cause a series of inflammatory cascade reactions, leading to immune disorders. The timing of targeted, personalized, and precise medical approaches to sepsis and whether sepsis or septic shock should be treated with empirical antibiotics are among the most important clinical priorities put forward by the sepsis Survival Research Committee [10]. In this case, early combined intervention including empirical antibiotic treatment is very important. CRRT, lavage and closed thoracic drainage stabilize the internal environment and earn valuable treatment time, which are significant for critically ill patients. It has been suggested in the literature that early, high-dose and long-term administration of intravenous immunoglobulin (IVIg) may improve the prognosis of patients [11]. The immunotherapy and nutritional support therapy we adopted also had a positive impact on the patient’s prognosis.

In conclusion, the present patient's condition was extremely critical when admitted, and the treatment was very difficult. He was cured and survived as a result of active comprehensive treatment and intervention. As each critical patient has his or her own features, this paper proposes a reference diagnosis and treatment solution for similar cases rather than a fixed scheme.

## Data Availability

The datasets used and/or analyzed during the current study are available from the corresponding author on reasonable request.

## Abbreviations

ARDS: Acute respiratory distress syndrome
CRRT: Continuous renal replacement therapy
IDSA/ATS: Infectious Diseases Society of America/American Thoracic Society
OI: Oxygenation index
PEEP: Positive end expiratory pressure
RICU: Respiratory intensive care unit
APACHEII: Acute Physiology and Chronic Health Evaluation II
WBC: White blood cells
PCT: Procalcitonin
CRP: C-reactive protein
BNP: Type B natriuretic peptide
eGFR: Estimated glomerular filtration rate
SBT: Spontaneous breathing experiments
RSBI: Rapid shallow breathing index
AKI: Acute renal injury
IVIg: Intravenous immunoglobulin
